# Short Chorus Wave Packets: Generation Within Chorus Elements, Statistics, and Consequences on Energetic Electron Precipitation

**DOI:** 10.1029/2022JA030310

**Published:** 2022-05-21

**Authors:** D. Mourenas, X.‐J. Zhang, D. Nunn, A. V. Artemyev, V. Angelopoulos, E. Tsai, C. Wilkins

**Affiliations:** ^1^ CEA DAM DIF Arpajon France; ^2^ Laboratoire Matière en Conditions Extrêmes Paris‐Saclay University CEA Paris France; ^3^ Department of Earth, Planetary, and Space Sciences University of California Los Angeles CA USA; ^4^ School of Electronics and Computer Science Southampton University Southampton UK

**Keywords:** chorus wave, nonlinear generation, wave superposition, wave beating, anomalous trapping, quasi‐linear diffusion

## Abstract

Short and intense lower‐band chorus wave packets are ubiquitous in the Earth's outer radiation belt. In this article, we perform various Vlasov hybrid simulations, with one or two triggering waves, to study the generation of short chorus packets/subpackets inside long rising tone elements. We show that the length of the generated short wave packets is consistent with a criterion of resonance non‐overlap for two independent superposed waves, and that these chorus packets have similar characteristics as in Van Allen Probes observations. We find that short wave packets are mainly formed near the middle/end of long rising tones for moderate linear growth rates, and everywhere for stronger linear growth rates. Finally, we analyze an event characterized by Time History of Events and Macroscale Interactions during Substorms spacecraft measurements of chorus rising tones near the equator and simultaneous measurements by low altitude ELFIN CubeSats of precipitating and trapped electron fluxes in the same sector. The measured precipitating electron fluxes are well recovered by test particle simulations performed using measured plasma and wave properties. We show that short chorus wave packets of moderate amplitudes (160–250 pT) essentially lead to a more diffusive‐like transport of 50–200 keV electrons toward the loss cone than long packets. In contrast, long chorus packets are found to produce important nonlinear effects via anomalous trapping, which significantly reduces electron precipitation below 150 keV, especially for higher wave amplitudes.

## Key Points

- Vlasov hybrid simulations consistently produce short chorus packets by wave superposition, with similar characteristics as in observations
- Short chorus wave packets are found in the middle/end of rising tones for low linear growth rates but everywhere for high growth rates
- Electron precipitation by short moderate amplitude chorus wave packets occurs in a nearly quasi‐linear regime

## 1 Introduction

Intense whistler‐mode chorus waves in the lower band frequency range (at *f* ≃ (0.1–0.5)*f*_*ce*_ with *f*_*ce*_ the equatorial electron gyrofrequency) are ubiquitous in the Earth's inner magnetosphere (Cattell et al., 2008; Cully et al., 2011; Li, Bortnik, et al., 2011; Meredith et al., 2001; Tsurutani & Smith, 1974; Zhang et al., 2019, 2018). The most intense waves are initially linearly excited with small wave‐normal angles near the magnetic equator by anisotropic electrons injected from the plasma sheet (Artemyev et al., 2016; Kennel, 1966; LeDocq et al., 1998; Li et al., 2010; Tsurutani & Smith, 1974). Next, nonlinear wave growth takes over, leading to the formation of intense rising tone chorus elements, typically lasting more than 100 ms (Demekhov & Trakhtengerts, 2008; Nogi & Omura, 2021; Nunn, 1974; Omura et al., 2008; Tao et al., 2017, 2021). During nonlinear wave growth, the geomagnetic field inhomogeneity controls the formation of an electron hole at cyclotron resonance with the wave (Karpman et al., 1974; Nunn, 1974) and the resulting resonant current leads to a simultaneous increase of wave amplitude and wave frequency, explaining the observed long rising tone elements (Demekhov et al., 2017; Omura et al., 2008; Summers et al., 2013). However, such long rising tone elements are usually composed of many subpackets of various amplitudes and durations (Cattell et al., 2008; Santolík et al., 2003, 2014; Tsurutani et al., 2020). Recent satellite statistics have indeed shown that most intense lower band chorus wave packets (or subpackets) in the inner magnetosphere are short, lasting less than 20 wave periods (i.e., less than ∼10 ms), with fast and random frequency and phase variations near their edges (Zhang, Agapitov, et al., 2020; Zhang et al., 2019; Zhang, Mourenas, et al., 2020; Zhang et al., 2018).

This fine structure of intense quasi‐parallel chorus waves has a significant impact on wave‐particle interactions. In particular, nonlinear trapping‐induced electron acceleration is substantially reduced as compared with the ideal case of a long coherent wave packet, leading to a more advective or diffusive‐like electron energization similar to an interaction with many isolated and independent short packets (Artemyev, Neishtadt, Vasiliev, et al., 2021; Kubota & Omura, 2018; Mourenas et al., 2018; Tao et al., 2013; Zhang, Agapitov, et al., 2020). Similarly, anomalous trapping of small pitch angle electrons near and within the loss cone, which can lead to an increase of their pitch angle slowing down precipitation (Albert et al., 2021; Artemyev, Neishtadt, Albert, et al., 2021; Gan et al., 2020; Kitahara & Katoh, 2019), is strongly reduced in the presence of a realistic fine structure consisting of short packets/subpackets (see Appendix in Mourenas et al., 2021). Therefore, the formation of short chorus wave packets/subpackets should be taken into account in models of chorus wave‐particle interaction in the outer radiation belt, to accurately describe electron nonlinear acceleration or microburst precipitation into the atmosphere (Artemyev, Neishtadt, Vasiliev, et al., 2021; Breneman et al., 2017; Chen et al., 2020; Kubota & Omura, 2018; Miyoshi et al., 2015; Mourenas et al., 2018; Tao et al., 2013; Zhang, Agapitov, et al., 2020).

Wave superposition (also called wave beating) has been noticed in various numerical simulations of chorus wave nonlinear generation using a sufficient initial anisotropy of the 5–50 keV electron population (Katoh & Omura, 2016; Kuzichev et al., 2019; Nunn et al., 2021; Zhang et al., 2021). A Bayesian analysis of a long rising tone chorus element measured by the Van Allen Probes has also indicated the simultaneous presence of different waves of similar amplitudes (Crabtree et al., 2017). Years‐long statistics of intense chorus wave packets (with peak amplitude *B*_*w*,*peak*_ > 50 pT) observed by the Van Allen Probes (Mauk et al., 2013) and Time History of Events and Macroscale Interactions during Substorms (THEMIS, see Angelopoulos, 2008) spacecraft further suggest the frequent presence of wave superposition, based on (a) the prevalence of short packets of length *β* < 10–20 wave periods (less than ∼10 ms), (b) the statistical characteristics of frequency variations near packet edges that correspond to characteristics of a simple model of superposition of two waves of slowly varying amplitudes, and (c) the existence of two separate spectral power peaks inside long packets (Zhang et al., 2019; Zhang, Mourenas, et al., 2020). Van Allen Probes statistics have revealed that chorus wave packet length increases like $\beta ∼B_{w,peak}^{3/2}$, with a frequency sweep rate scaling like *∂f*/*∂t* ∼ *f* ^2^/*β* ^2^ (Nunn et al., 2021; Zhang et al., 2021). These two characteristics of chorus wave packets have been well reproduced by numerical simulations (Nunn et al., 2021; Zhang et al., 2021), lending confidence in numerical simulations as a practical laboratory for a detailed investigation of chorus wave packet formation.

A first analysis of chorus wave packet formation with the Vlasov hybrid simulation (VHS) code (Nunn, 2005; Nunn et al., 2009) has shown that using two triggering waves in a simulation to favor the generation of simultaneous chorus waves, leads to the formation of short packets with similar statistical properties as in satellite observations (Nunn et al., 2021). Based on theoretical considerations, the formation in this VHS simulation of not‐too‐short high‐amplitude packets/subpackets has been attributed to nonlinear trapping‐induced wave amplitude modulation (Demekhov & Trakhtengerts, 2008; Omura et al., 2008; Tao et al., 2017), whereas the formation of short and moderate amplitude packets/subpackets has been ascribed to wave superposition constrained by the Chirikov criterion for resonance non‐overlap of two independent nonlinearly generated waves, which requires a sufficient frequency difference (Nunn et al., 2021).

However, the VHS simulation of short packet formation analyzed by Nunn et al. (2021) was performed with two triggering waves and for a given set of initial conditions. We still need to check whether short chorus wave packets can be formed in VHS simulations for significantly different initial conditions, especially in the presence of only one triggering wave. In the present work, we first present in Section 2 the present status of theories of short chorus wave packet generation. Next, we investigate in Section 3, the formation of short chorus packets with the VHS code with new (different) initial conditions not investigated in Nunn et al. (2021) and compare the resulting wave packet statistics, obtained through a new method of analysis, with both theory and observations, strengthening the conclusions of Nunn et al. (2021) concerning the origin of short wave packets. We complete this study by a novel analysis of the temporal localization of short packets inside long chorus rising tone elements in VHS simulations, to check whether they occur in a particular part (start, middle, or end) of a long rising tone. Finally, we investigate in Section 4 a selected event with THEMIS spacecraft observations of successive long chorus rising tones at *L*‐shell (*L*) around 6. The characteristics of chorus wave packets obtained during this event by THEMIS near the magnetic equator are used to evaluate for the first time via test particle simulations the consequences of the fine structure of intense chorus waves on energetic electron precipitation into the atmosphere. The results of such test particle simulations are compared with nearly conjugate observations of electron precipitation by the low altitude ELFIN CubeSat (Angelopoulos et al., 2020) during the same event.

## 2 Generation of Short Chorus Wave Packets: Theoretical Models

Based on theory, short chorus wave packets can be produced either by nonlinear trapping‐induced wave amplitude modulation, or by wave superposition, also called wave beating (e.g., see Nunn et al., 2021, and references therein). Let us briefly examine below these two types of models.

### 2.1 Short Packet Formation by Trapping‐Induced Amplitude Modulation

The nonlinear modulation of the amplitude of an intense single wave at the trapping frequency *ω*_*tr*_ of cyclotron resonant electrons can form wave packets of length *β*_*tr*_ = *ω*/*ω*_*tr*_ (Morales & O’Neil, 1972; Nunn et al., 2021; Tao et al., 2017, 2021; Trakhtengerts et al., 2004), with

(1) $$\beta_{tr}\;≃\;\frac{\gamma^{1/2}\;\left(\frac{\omega }{\Omega_{ce}}\right){\left(\frac{\Omega_{ce}}{\omega }-1\right)}^{1/4}}{{\left[\frac{V_{⊥}\;\Omega_{pe}\;B_{w,peak}}{c\;\;\Omega_{ce}\;B_{0}}\right]}^{1/2}},$$

with *ω* the average wave frequency, Ω_*ce*_ the electron gyrofrequency, Ω_*pe*_ the electron plasma frequency, γ the Lorentz factor, *B*_*w*,*peak*_ the wave packet peak amplitude, *B*_0_ the geomagnetic field strength, *V*_⊥_ the transverse velocity of cyclotron resonant electrons, and *c* the speed of light.

We consider typical wave and plasma parameters near the magnetic equator at 0–6 MLT and *L* ∼ 4–6 outside the plasmasphere, namely, an electron plasma frequency to gyrofrequency ratio Ω_*pe*_/Ω_*ce*_ ∼ 3–5 (Carpenter & Anderson, 1992; Sheeley et al., 2001), quasi‐parallel chorus wave frequency *ω*/Ω_*ce*_ ∼ 0.2–0.5 (independently of *β* in Van Allen Probes statistics), and a realistic transverse energy *E*_⊥_ ≤ 100 keV of the most abundant cyclotron resonant electrons (injected from the plasma sheet) providing the free energy for wave growth (Agapitov et al., 2018; Li et al., 2016, 2010). For such typical parameters in the outer radiation belt, Equation 1 gives wave packet lengths *β*_*tr*_ larger than a minimum length:

(2) $$\beta_{tr,\min\;}≃0.2\;{\left(\frac{B_{0}}{B_{w,peak}}\right)}^{1/2},$$

This leads to a minimum length *β*_*tr*, min_ ∼ 10 (corresponding to more than ∼5–10 ms) for packets with *B*_*w*,*peak*_ ≤ 100 pT produced by trapping‐induced wave amplitude modulation. Tao et al. (2021) further pointed out that the trapping period measured at a fixed location should be (1 − *v*_*res*_/*v*_*g*_) ∼ (1 + Ω_*ce*_/2*ω*) times longer than 2*π*/*ω*_*tr*_ (with *v*_*res*_ the cyclotron resonant electron velocity and *v*_*g*_ the wave group velocity; see also Dowden, 1982; Nunn, 1986), corresponding to an increased minimum length

(3) $$\beta_{tr,min2}≃0.65\;{\left(\frac{B_{0}}{B_{w,peak}}\right)}^{1/2}.$$

for packets produced by trapping‐induced wave amplitude modulation.

The nonlinear generation of intense chorus waves near the magnetic equator is accompanied by a nonlinear frequency sweep rate $|\partial f/\partial t{|}_{NL}≃0.16\;\omega^{2}|S|/{\left[\beta_{tr}\left(1+\Omega_{ce}/2\omega \right)\right]}^{2}$, producing rising or falling tones (Cully et al., 2011; Macúšová et al., 2010; Nunn, 1974; Nunn et al., 2009; Omura et al., 2008; Shklyar & Matsumoto, 2009; Vomvoridis et al., 1982). The inhomogeneity factor *S* must satisfy the inequality |*S*| < 1 to get electron trapping and nonlinear wave growth (Omura et al., 2008). It is generally taken as |*S*| ∼ 0.4–0.5 to maximize electron‐wave energy transfer (Omura et al., 2008; Tao et al., 2021; Vomvoridis et al., 1982), giving an approximate upper limit |*∂f*/*∂t*|_*NL*_[kHz/s] < 0.2 *B*_*w*,*peak*_[pT] at *L* ∼ 5 (Nunn et al., 2021).

### 2.2 Short Packet Formation by Wave Superposition

Statistics from the Van Allen Probes indicate that most of the observed chorus packets/subpackets have moderate amplitudes *B*_*w*,*peak*_ ∼ 70–200 pT, are shorter than *β*_*tr*, min_ ∼ *β*_*tr*,*min*2_/3 ≈ 10, and have huge frequency sweep rates |*∂f*/*∂t*| ∼ 50–400 kHz/s > |*∂f*/*∂t*|_*NL*_ (Zhang, Mourenas, et al., 2020). Such short packets/subpackets cannot be formed by nonlinear electron trapping by a single wave. However, they can be produced by the beating of two waves (of frequencies *ω*_1_ and *ω*_2_) of similar amplitudes, leading to an amplitude modulation at their frequency difference Δ*ω* = *ω*_2_ − *ω*_1_ (Nunn et al., 2021; Tao et al., 2013; Zhang, Mourenas, et al., 2020). Simulations from various codes show that two separate, independent chorus waves can indeed grow nonlinearly at the same time near the geomagnetic equator in the presence of a realistic, anisotropic electron population, producing short wave packets with similar characteristics as in Van Allen Probes statistics (Katoh & Omura, 2016; Nunn et al., 2021; Zhang et al., 2021).

In the case of a too small frequency difference Δ*ω* between two independent waves, however, their resonances can overlap and lead to a stochastization of electron trajectories (Chirikov, 1979), perturbing the nonlinear resonant current (Omura et al., 2013) of the weaker wave and preventing its independent nonlinear growth to a significant amplitude (Nunn et al., 2021). Consequently, Nunn et al. (2021) suggested that two *independent* quasi‐parallel chorus waves can grow nonlinearly and reach significant amplitudes only if the corresponding two cyclotron resonances are non‐overlapping, corresponding to a frequency difference Δ*ω* larger than a minimum value Δ*ω*_min_. For typical conditions Ω_*pe*_/Ω_*ce*_ ≫ 1 at *L* = 4–6 and γ ∼ 1, this minimum frequency separation for resonance non‐overlap can be written as Δ*ω*_min_ ≃ 2^3/2^ *ω*_*tr*_/(1 + Ω_*ce*_/2*ω*) for two waves of similar amplitudes equal to half the total wave amplitude *B*_*w*_ = *B*_*w*1_ + *B*_*w*2_ ∼ 2*B*_*w*1_ (Nunn et al., 2021; Omura, 2021).

Both numerical simulations (Omura et al., 2008; Tao et al., 2017) and comparisons of cyclotron resonance diffusion surfaces and constant energy surfaces (Horne & Thorne, 2003; Summers et al., 1998) indicate that the free energy for chorus wave growth mainly comes from electrons with a transverse to parallel momentum ratio *p*_⊥_/*p*_∥_ > 1. Using the cyclotron resonance condition $p_{∥,R}=m_{e}c\;\left(\Omega_{ce}/\Omega_{pe}\right){\left(\Omega_{ce}/\omega \right)}^{1/2}{\left(1-\omega /\Omega_{ce}\right)}^{3/2}$ and substituting *p*_⊥_ = *p*_∥,*R*_ in the expression of Δ*ω*_min_ then gives an estimate of the minimum frequency separation allowing a substantial growth of two simultaneous, independent waves of similar amplitudes (Nunn et al., 2021):

(4) $$\frac{{\Delta \omega }_{\min}}{\omega }=2^{5/2}\;{\left(\frac{B_{w,peak}}{B_{0}}\right)}^{1/2}\frac{\Omega_{ce}^{1/2}{\left(\Omega_{ce}-\omega \right)}^{1/2}}{2\;\omega +\Omega_{ce}}.$$

For typical lower‐band chorus packets with *B*_*w*,*peak*_ ∼ 100 pT at *L* ∼ 5–6 (Zhang, Mourenas, et al., 2020), Equation 4 yields Δ*ω*_min_/*ω* ≃ 0.06–0.1, in agreement with minimum frequency differences obtained in Fast Fourier Transform (FFT) spectra of long chorus packets and with minimum frequency differences inferred from fast frequency variations within observed chorus packets (Zhang, Mourenas, et al., 2020). A superposition of two independent waves of similar amplitudes separated by a frequency difference Δ*ω* > Δ*ω*_min_ can lead to the formation of short chorus wave packets of length *β*_*ws*_ < *β*_*ws*, max_ = *ω*/Δ*ω*_min_, with:

(5) $$\beta_{ws,\max\;}≃0.5\;{\left(\frac{B_{0}}{B_{w,peak}}\right)}^{1/2}≃\frac{\beta_{tr,min2}}{1.3}.$$

Equation 5 shows that packets formed by wave superposition should be shorter than *β*_*ws*, max_ ≃ *β*_*tr*,*min*2_/1.3 and can also be shorter than *β*_*tr*, min_. A large majority of the observed chorus wave packets/subpackets and nearly all moderate amplitude packets with |*∂f*/*∂t*| ∼ 50–400 kHz/s > |*∂f*/*∂t*|_*NL*_, satisfy the condition *β* < *β*_*ws*, max_, with nearly as many short packets with negative and positive frequency sweep rates and $|\partial f/\partial t{|}_{ws}∼{\left(\Delta \omega_{ws}\right)}^{2}∼f^{2}/\beta_{ws}^{2}$, as expected for a superposition of two independent waves of slowly varying amplitudes (Zhang, Mourenas, et al., 2020). In the presence of wave superposition, random jumps in frequency and phase between successive packets tend to detrap resonant electrons and hamper nonlinear wave growth, possibly accounting for their generally moderate amplitudes *B*_*w*,*peak*_ ∼ 70–200 pT (Zhang, Agapitov, et al., 2020; Zhang, Mourenas, et al., 2020).

Lastly, although so‐called sideband waves can grow spontaneously very close to a main (sufficiently intense) wave in an inhomogeneous magnetic field (Costabile et al., 2017; Nunn, 1974, 1986), it is worth noting that (a) such sideband waves are not generated independently from the main wave but are instead directly generated by cyclotron resonance with particles trapped by the main wave (they are a by‐product of the nonlinear growth of this main wave), and (b) their growth rate is largest for a frequency difference with the main wave Δω_*_ ≃ *ω*_*tr*_/(1 − *v*_*res*_/*v*_*g*_) ≃ *ω*_*tr*_/(1 + Ω_*ce*_/2*ω*) (Dowden, 1982; Nunn, 1986). Accordingly, a sideband wave reaching a sufficiently high amplitude, similar to the main wave amplitude, is expected to produce wave amplitude modulations corresponding to relatively long packets with β_*_ = *ω*/Δω_*_ > *β*_*tr*,*min*2_ > *β*_*tr*, min_, similar to the trapping‐induced modulations already considered in Section 2.1.

## 3 Simulations of Short Chorus Packet Generation: Dependence on Initial Conditions and Comparisons With Statistical Observations

In the present study, as in previous works (Nunn et al., 2021; Zhang et al., 2021; Zhang, Mourenas, et al., 2020), lower‐band chorus wave packets are identified by a peak of full wave amplitude *B*_*w*,*peak*_ above 50 pT and the packet boundaries are set at the nearest *B*_*w*_ minimum below 50 pT, or else at the time when *B*_*w*_ diminishes to 10 pT. The selected wave packets can be either isolated wave packets, or subpackets located inside a long rising tone chorus element, or much more rarely, long rising tone packets only weakly modulated on shorter time scales. The packet length *β* is the number of wave periods inside a packet. The average frequency sweep rate *∂f*/*∂t* within a packet is calculated through linear regression, based on wave half‐periods between successive zero crossings of one transverse component of the wave amplitude (Zhang, Mourenas, et al., 2020).

Using a VHS code (Nunn, 2005, 2021), one‐dimensional along the inhomogeneous magnetic field as appropriate for parallel propagating waves growing from the equator, Nunn et al. (2021) have shown that using two triggering waves of small amplitudes generates many short packets by forcing the initially unstable plasma system to produce wave superpositions. However, the results from Nunn et al. (2021) were mostly obtained for a specific set of initial conditions. Here, we examine new (different) initial conditions: one more simulation with two triggering waves and different initial parameters, as well as two other simulations with only one triggering wave. This allows us to produce different types of long (∼50–150 ms) chorus elements and to check when and where the short wave packets are produced inside these long chorus elements. Note that in the work of Nunn et al. (2021), only wave packets with 〈*f*〉 > 2.45 kHz were kept in their so‐called “one‐wave simulation”, to exclude short packets formed by a significant wave superposition present at 1.9–2.4 kHz in their Figure S3(top). This enabled a comparison between a “one‐wave simulation” (nearly without wave superposition) and a “two‐wave simulation” (with significant wave superposition) in their Figure 3, with similar *B*_*w*,*peak*_ distributions in both data sets, resulting in clear statistical differences. However, the “one‐wave simulation” from Nunn et al. (2021) still contained a few periods of wave superposition.

**Figure 1:**
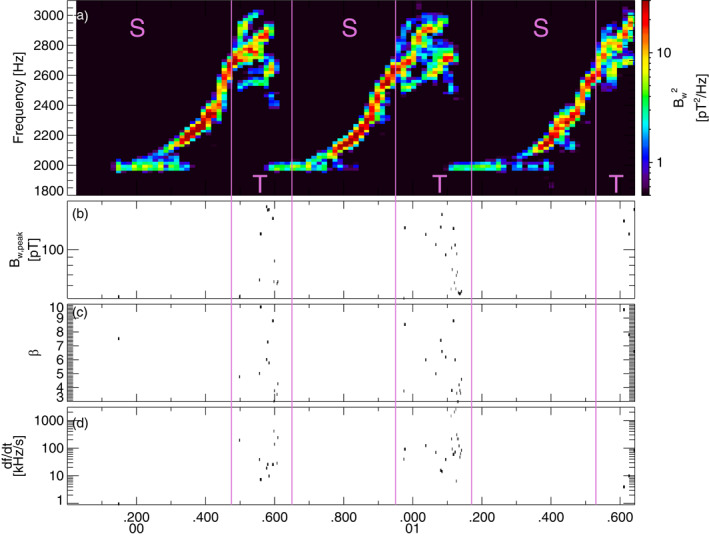
(a) Lower‐band chorus wave Fast Fourier Transform (FFT) spectrum (using 256‐point FFT windows with 30% overlap between adjacent windows) from the first Vlasov hybrid simulation (VHS) simulation with one triggering wave, obtained 1,345 km away from the magnetic equator. Intervals with only one single wave are marked by an S, intervals with two (or more) superposed waves are marked by a T, and these intervals are separated by vertical purple lines. (b) Peak amplitude of identified short packets, of length *β* < 10. (c) Length *β* of short packets. (d) Frequency sweep rate *∂f*/*∂t* of short packets.

To improve our analysis of wave packet formation as compared with previous studies (Nunn et al., 2021; Zhang et al., 2021) where each simulation run was analyzed as a whole, we hereafter carefully split the results of each simulation into Two Waves intervals (labeled T) and Single Wave intervals (labeled S). Two waves intervals contain a superposition of one main wave of amplitude *B*_*w*1_ and one or more other waves, separated from the main wave by Δ*f*/*f* > 0.06, with similar individual amplitude *B*_*w*2_ (or total amplitude *B*_*w*2_ = *∑B*_*w*2,*i*_) such that 0.5 ≤ *B*_*w*2_/*B*_*w*1_ ≤ 2. Single Wave intervals contain no such wave superposition. These different intervals are first identified in the FFT spectrum (e.g., see Figure 1a), and the two types of intervals are later analyzed separately, to more accurately determine their statistical differences.

**Figure 2:**
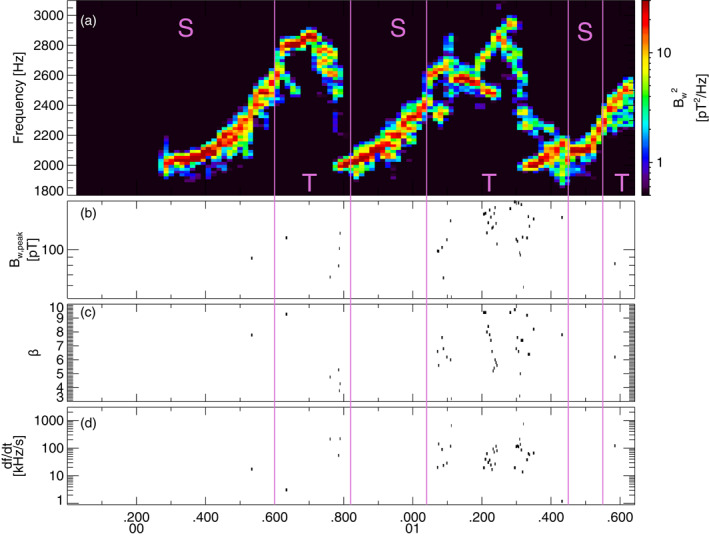
(a) Lower‐band chorus wave Fast Fourier Transform (FFT) spectrum from the first Vlasov hybrid simulation (VHS) simulation with one triggering wave, obtained 6,000 km away from the magnetic equator. Intervals with only one single wave are marked by an S, intervals with two (or more) superposed waves are marked by a T, and these intervals are separated by vertical purple lines. (b) Peak amplitude of identified short packets, of length *β* < 10. (c) Length *β* of short packets. (d) Frequency sweep rate *∂f*/*∂t* of short packets.

In the present VHS simulations, one or two keydown (constant) triggering waves with small amplitudes are introduced at *z* = −6,000 km in a simulation box going from *z* = −6,000 km to *z* = +6,000 km (Nunn, 2021). Such triggering waves are externally imposed in the simulation (with a constant frequency and a small amplitude), each triggering wave constituting a seed for subsequent nonlinear rising tone chorus generation at slightly higher frequencies by the unstable anisotropic electron distribution (Nunn et al., 2021). Realistic conditions in the outer radiation belt at *L* ∼ 5 are used, with electron gyrofrequency *f*_*ce*_ = 6.7 kHz, cold plasma density *N*_*e*_ = 5.4 cm^−3^ outside the plasmasphere, and two hot electron populations. This plasma density level is typical at 0–6 MLT near *L* = 5 outside the plasmasphere (Carpenter & Anderson, 1992; Sheeley et al., 2001) and gives a ratio Ω_*pe*_/Ω_*ce*_ ∼ 3.1 at *L* = 5—the average *L*‐shell of Van Allen Probes chorus wave statistics covering *L* = 4–6 (Zhang et al., 2018).

In the first simulation, one 6 pT keydown triggering wave is used. The zero order distribution function consists of two bi‐Maxwellians. The lower energy one has temperatures *T*_⊥_ = 44 keV and *T*_∥_ = 15 keV, and the higher energy one has *T*_⊥_ = 192 keV and *T*_∥_ = 60 keV. Both have realistic anisotropies *A* ∼ 2 and are thus linearly unstable (Li et al., 2010). Both bi‐Maxwellians are normalized to unity, and the hottest distribution is weighted by a factor 26.3, to give a realistic overall roll‐off with energy. The combined distribution function is then re‐normalized to obtain a linear growth rate in the simulation of 130 dB/s at the equator at the base triggering wave frequency *ω* = 0.3Ω_*ce*_ (or *f* = 2.01 kHz), coinciding with triggered chorus waves. The resulting chorus wave frequencies are typical of lower‐band chorus waves observed near the equator (Agapitov et al., 2018) and correspond to cyclotron resonant electrons with parallel energy ≃ 15–50 keV.

FFT spectra from the first simulation with one keydown triggering wave inside the chorus generation region are provided in top panels of Figures 1 and 2 near and slightly away from the magnetic equator, respectively. Close to the equator, the first part of each long rising tone chorus element corresponds to a Single Wave interval, whereas the second part of each element corresponds to a Two Waves interval, with a superposition of different waves simultaneously generated above and below the primary wave of increasing frequency when it increases above ∼0.4*f*_*ce*_. These additional waves are probably generated by the nonlinear perturbations of the hot electron distribution caused by the primary wave. Many short packets of length *β* = 4–10 (corresponding to ∼1.5–5 ms), peak amplitudes *B*_*w*,*peak*_ = 60–200 pT, and large frequency sweep rates |*∂f*/*∂t*| ∼ 30–1,000 kHz/s are formed in the second part of each long chorus rising tone element but, remarkably, none in the first part of each element. In the second part of each rising tone chorus element, wave superposition leads to amplitude modulation and formation of packets of length *β* ∼ *ω*/Δ*ω* (Nunn et al., 2021; Tao et al., 2013). The long rising tone elements have average frequency sweep rates |*∂f*/*∂t*| ∼ 5 kHz/s in agreement with the nonlinear sweep rate |*∂f*/*∂t*|_*NL*_. Farther away from the equator, some short packets start to appear in the first part of each long chorus element, due to a superposition of the low‐frequency start of a chorus element with the high‐frequency end of the preceding chorus element, which often shows hook‐like features. The long chorus elements obtained in this simulation are quite realistic, since very similar chorus rising tones have been observed by the Van Allen Probes around *L* ∼ 5–6 (Foster et al., 2021).

**Figure 3:**
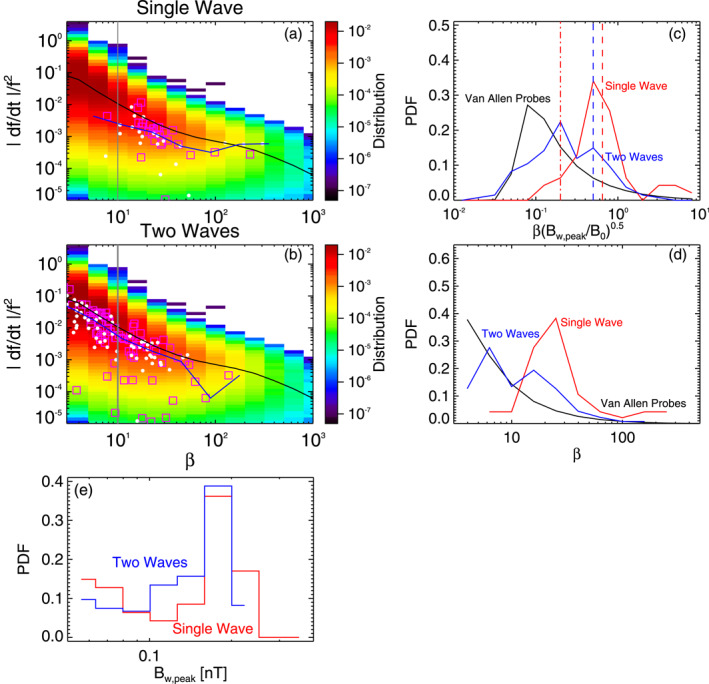
(a) Vlasov hybrid simulation (VHS) code results from the first simulation with one triggering wave, during Single Wave intervals in Figures 1 and 2. Normalized frequency sweep rate |*∂f*/*∂t*|/*f* ^2^ of wave packets obtained in the simulation, within the generation region close to the equator (white circles) and 6,000 km away from the equator (magenta squares), as a function of packet length *β* (the median is shown by a blue curve). Corresponding statistical results from Van Allen Probes 2012–2018 observations are displayed in colors, with their median |*∂f*/*∂t*|/*f* ^2^ shown by a black curve. A gray line shows *β* = 10. (b) Same as (a) during Two Waves intervals. (c) Probability Distribution Function of wave packets as a function of $\beta \;{\left(B_{w,peak}/B_{0}\right)}^{1/2}$ obtained from Van Allen Probes statistical observations (black curve) and from the VHS code simulation during Single Wave intervals (red) and Two Waves intervals (blue). The minimum lengths *β*_*tr*, min_ and *β*_*tr*,min2_ from Equations 2 and 3 of wave packets formed by nonlinear trapping‐induced wave amplitude modulation alone are shown by dashed‐dotted and dashed red vertical lines, respectively. The maximum length *β*_*ws*, max_ from Equation 5 of packets formed by wave superposition alone is indicated by a dashed blue vertical line. (d) Probability distributions of wave packets from Van Allen Probes statistical observations (black) and from the VHS code simulation during Single Wave intervals (red) and Two Waves intervals (blue), as a function of packet length *β*. (e) Probability distributions of wave packets from the VHS code simulation during Single Wave intervals (red) and Two Waves intervals (blue), as a function of packet peak amplitude *B*_*w*,*peak*_.

Figure 3 shows statistical results from the first VHS simulation with one keydown triggering wave, compared with statistical results from 6 yr of Van Allen Probes observations of quasi‐parallel lower‐band chorus wave packets at *L* ≃ 4.5–5.5. Wave packets from Van Allen Probes observations are determined in the same way as packets from the simulation. The Probability Distribution Functions (PDFs) of the peak amplitude *B*_*w*,*peak*_ of wave packets obtained in the simulation during Single Wave intervals (in red) and Two Waves intervals (in blue) are similar in Figure 3e, both extending from 60 to 220 pT.

The PDFs of wave packets obtained during Single Wave intervals (in red) of the simulation are strikingly different from the PDFs of chorus packets from statistical Van Allen Probes observations (in black) in Figures 3c and 3d, since the latter peak at low *β* < 10 and $\beta \;{\left(B_{w,peak}/B_{0}\right)}^{1/2}<0.25$, whereas the former have a high maximum at *β* = 15–30 and $\beta \;{\left(B_{w,peak}/B_{0}\right)}^{1/2}=0.4-1.0$. In contrast, the PDFs of wave packets obtained during Two Waves intervals (in blue) of the simulation are very similar to the PDFs of chorus packets from statistical Van Allen Probes observations, with similar shapes and a similar peak of occurrence at a low value of *β* and $\beta \;{\left(B_{w,peak}/B_{0}\right)}^{1/2}$. This suggests that most packets in Van Allen Probe observations are likely due to wave superposition, as in Two Wave intervals of the simulation.

In particular, Figures 3a and 3d demonstrate that Single Wave intervals contain only a very small percentage ∼5% of short wave packets with *β* < 10 (less than ∼5 ms). In contrast, nearly 50% of the chorus packets obtained during Two Waves intervals are short (with *β* < 10). Figures 3a and 3c show that during Single Wave intervals in the simulation, ∼50% of the packets have lengths *β* ≥ *β*_*tr*,*min*2_ and ∼95% of the packets have lengths *β* > *β*_*tr*, min_, which can result from the sole wave amplitude modulation produced by nonlinear electron trapping in a single wave potential (Morales & O’Neil, 1972). During such Single Wave intervals, the shortest packets are probably still mainly formed by trapping‐induced amplitude modulation, but with the help of an additional, weaker amplitude modulation due to a superposition of the main wave with a wave of much smaller amplitude (as near 0.53 s in Figure 2). On the other hand, Figure 3c shows that during Two Waves intervals of the simulation, ∼80% of the packets have lengths *β* ≤ *β*_*ws*, max_ that can be produced by wave superposition. Moreover, ∼50% of the Two Waves interval packets have lengths *β* < *β*_*tr*, min_ and ∼80% have lengths *β* < *β*_*tr*,*min*2_, showing that at least half of these packets cannot result from trapping‐induced amplitude modulation alone. Normalized frequency sweep rates |*∂f*/*∂t*|/*f* ^2^ of wave packets in Figures 3a and 3b have a similar scaling |*∂f*/*∂t*|/*f* ^2^ ∼ 1/*β* ^2^ in observations and simulation, in agreement with the similar dependencies on *β* produced by nonlinear effects (Demekhov & Trakhtengerts, 2008; Nunn et al., 2009; Omura et al., 2008) and wave superposition (Nunn et al., 2021; Zhang et al., 2021; Zhang, Mourenas, et al., 2020).

**Figure 4:**
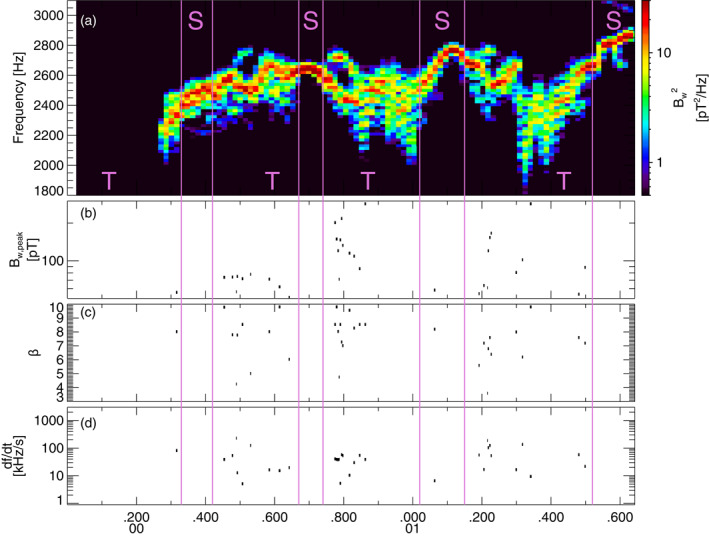
(a) Lower‐band chorus wave Fast Fourier Transform (FFT) spectrum from the second Vlasov hybrid simulation (VHS) simulation with one triggering wave, obtained within 6,000 km of the magnetic equator. Intervals with only one single wave are marked by an S, intervals with two (or more) superposed waves are marked by a T, and these intervals are separated by vertical purple lines. (b) Peak amplitude of identified short packets, of length *β* < 10. (c) Length *β* of short packets. (d) Frequency sweep rate *∂f*/*∂t* of short packets.

The parameters of the second VHS simulation are exactly the same as for the first simulation, except that the densities of the two bi‐Maxwellian hot electron populations are increased by a factor of 1.85. Accordingly, the maximum linear growth rate of ∼240 dB/s is nearly twice larger than in the first simulation, just above the frequency (2.01 kHz) of the unique 6 pT keydown triggering wave. Figure 4 shows that chorus elements produced by this second simulation have very different shapes compared with the first simulation in Figures 1 and 2, with much less clear and well‐separated rising tones and many more hooks or falling tone portions. Previous studies have indeed shown that when the linear drive of chorus waves becomes sufficiently strong, the optimum wave amplitude becomes much larger than the threshold amplitude for nonlinear growth (Omura & Nunn, 2011), leading to the generation of more closely located and less distinguishable chorus elements (Katoh & Omura, 2013; Tao et al., 2020). Figure 4 shows that Single Wave intervals are now mainly located in the middle or end of long rising tone elements, while Two Waves intervals can be encountered anywhere inside all long chorus elements, corresponding to the formation of many short packets with *β* = 4–10.

**Figure 5:**
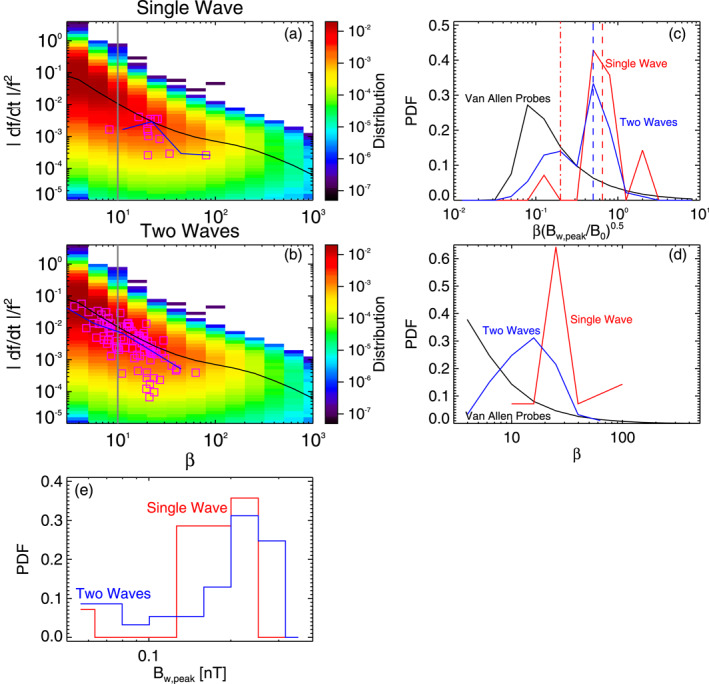
(a) Vlasov hybrid simulation (VHS) code results from the second simulation with one triggering wave, during Single Wave intervals in Figure 4. Normalized frequency sweep rate |*∂f*/*∂t*|/*f* ^2^ of wave packets obtained in the simulation approximately 6,000 km away from the equator (magenta squares) as a function of packet length *β* (the median is shown by a blue curve). Corresponding statistical results from Van Allen Probes 2012–2018 observations are displayed in colors, with their median |*∂f*/*∂t*|/*f* ^2^ shown by a black curve. A gray line shows *β* = 10. (b) Same as (a) during Two Waves intervals. (c) Probability Distribution Function of wave packets as a function of $\beta \;{\left(B_{w,peak}/B_{0}\right)}^{1/2}$ obtained from Van Allen Probes statistical observations (black curve) and from the VHS code simulation during Single Wave intervals (red) and Two Waves intervals (blue). The minimum lengths *β*_*tr*, min_ and *β*_*tr*,min2_ from Equations 2 and 3 of wave packets formed by nonlinear trapping‐induced wave amplitude modulation alone are shown by dashed‐dotted and dashed red vertical lines, respectively. The maximum length *β*_*ws*, max_ from Equation 5 of packets formed by wave superposition alone is indicated by a dashed blue vertical line. (d) Probability distributions of wave packets from Van Allen Probes statistical observations (black) and from the VHS code simulation during Single Wave intervals (red) and Two Waves intervals (blue), as a function of packet length *β*. (e) Probability distributions of wave packets from the VHS code simulation during Single Wave intervals (red) and Two Waves intervals (blue), as a function of packet peak amplitude *B*_*w*,*peak*_.

Figure 5 shows statistical results from the second VHS simulation, compared with statistical observations from the Van Allen Probes. PDFs of wave packet peak amplitudes *B*_*w*,*peak*_ obtained in the simulation during Single Wave intervals (in red) and Two Wave intervals (in blue) are similar in Figure 5e, and roughly similar to first simulation's results in Figure 3e. In Figures 5c and 5d, the PDFs of wave packets obtained during Single Wave intervals (in red) of the simulation are again very different from the PDFs of chorus packets from statistical Van Allen Probes observations (in black): the latter peak at low *β* < 10 (corresponding to less than ∼4–5 ms) and $\beta \;{\left(B_{w,peak}/B_{0}\right)}^{1/2}<0.25$, whereas the former have a maximum at *β* = 25 and $\beta {\left(B_{w,peak}/B_{0}\right)}^{1/2}=0.5-9$. However, PDFs of wave packets obtained during Two Waves intervals of the simulation (in blue) are significantly closer to PDFs of chorus packets from statistical Van Allen Probes observations, with occurrences shifted toward smaller *β* and $\beta \;{\left(B_{w,peak}/B_{0}\right)}^{1/2}$ values as compared with PDFs from Single Wave intervals.

Figures 5a and 5d further show that ∼93% of packets during Single Wave intervals are long (with *β* > 10), whereas nearly 60% of the packets identified during Two Waves intervals are short (with *β* < 10). During Single Wave intervals of the simulation, 51% of the packets have lengths *β* > *β*_*tr*,*min*2_ and 92% of the packets have lengths *β* > *β*_*tr*, min_, likely resulting from nonlinear trapping‐induced amplitude modulation of a single wave. Conversely, during Two Waves intervals ∼65% of the packets have lengths *β* ≤ *β*_*ws*, max_ that can be produced by wave superposition (see Figure 5c). Roughly ∼35% of Two Waves interval packets have lengths *β* < *β*_*tr*, min_ and ∼77% have lengths *β* < *β*_*tr*,*min*2_, implying that many of these packets cannot be produced by trapping‐induced amplitude modulation. Normalized sweep rates |*∂f*/*∂t*|/*f* ^2^ of wave packets show a similar dependence |*∂f*/*∂t*|/*f* ^2^ ∼ 1/*β* ^2^ in observations and simulation in Figures 5a and 5b, in agreement with dependencies expected from nonlinear effects and wave superposition.

**Figure 6:**
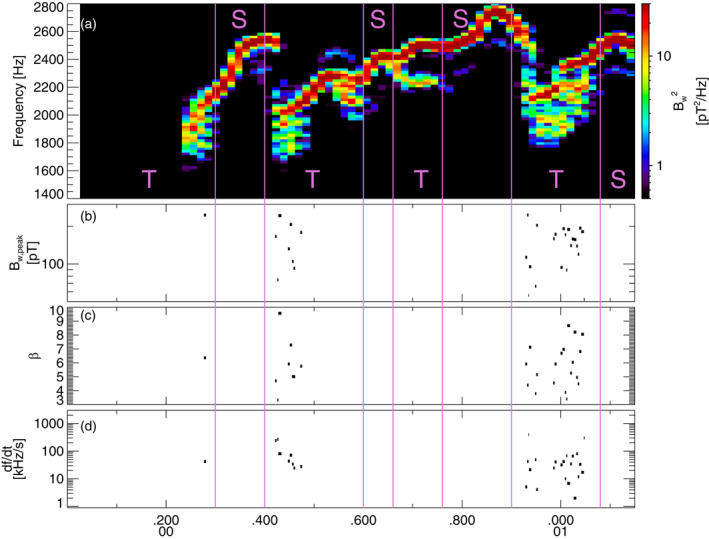
(a) Lower‐band chorus wave Fast Fourier Transform (FFT) spectrum from the third Vlasov hybrid simulation (VHS) simulation, with two triggering waves, obtained approximately 6,000 km away from the magnetic equator. Intervals with only one single wave are marked by an S, intervals with two (or more) superposed waves are marked by a T, and these intervals are separated by vertical purple lines. (b) Peak amplitude of identified short packets, of length *β* < 10. (c) Length *β* of short packets. (d) Frequency sweep rate *∂f*/*∂t* of short packets.

In the third VHS simulation, we use two 10 pT keydown triggering waves at *ω*_1_ = 0.26Ω_*ce*_ (*f* = 1.76 kHz) and *ω*_2_ = 0.3Ω_*ce*_. The perpendicular and parallel temperatures of the two bi‐Maxwellian hot electron populations, normalized to unity, are changed to *T*_⊥_ = 84  and *T*_∥_ = 25 keV for the lower energy bi‐Maxwellian, and *T*_⊥_ = 200  and *T*_∥_ = 60 keV for the higher energy one, which is now weighted by a factor 26.0. The combined distribution function is finally re‐normalized to give a linear growth rate of 180 dB/s (at 2.01 kHz at the equator), that is, higher than in the first simulation but smaller than in the second simulation. This third simulation produces long chorus elements of an intermediate shape as compared with the two other simulations. Note that we use a realistic frequency difference between triggering waves Δ*ω*/*ω* ≃ 1/6 > Δ*ω*_min_/*ω*, in agreement with typical frequency differences in chorus wave observations (Crabtree et al., 2017; Li, Thorne, et al., 2011; Zhang, Mourenas, et al., 2020) and realistic simulations (Katoh & Omura, 2016). Such a frequency difference should allow two independent triggered chorus waves to separately trap resonant particles, grow nonlinearly, and subsequently form short packets via wave beating (Nunn et al., 2021). In Figure 6, Single Wave intervals are generally located in the middle or end of long rising tone elements. Distinct rising or falling tone parts are sometimes simultaneously present, corresponding to Two Waves intervals near start, middle, or end of long elements, leading to the formation of short packets with *β* = 4–9 (lasting roughly ∼2–4 ms).

**Figure 7:**
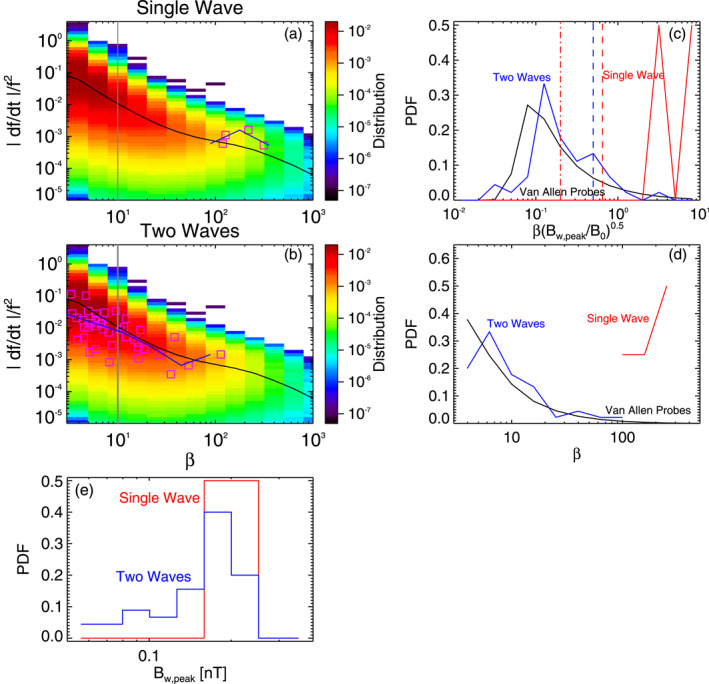
(a) Vlasov hybrid simulation (VHS) code results from the third simulation, with two triggering waves, during Single Wave intervals in Figure 6. Normalized frequency sweep rate |*∂f*/*∂t*|/*f* ^2^ of wave packets obtained in the simulation approximately 6,000 km away from the equator (magenta squares), as a function of packet length *β* (the median is shown by a blue curve). Corresponding statistical results from Van Allen Probes 2012–2018 observations are displayed in colors, with their median |*∂f*/*∂t*|/*f* ^2^ shown by a black curve. A gray line shows *β* = 10. (b) Same as (a) during Two Waves intervals. (c) Probability Distribution Function of wave packets as a function of $\beta \;{\left(B_{w,peak}/B_{0}\right)}^{1/2}$ obtained from Van Allen Probes statistical observations (black curve) and from the VHS code simulation during Single Wave intervals (red) and Two Waves intervals (blue). The minimum lengths *β*_*tr*, min_ and *β*_*tr*,*min*2_ from Equations 2 and 3 of wave packets formed by nonlinear trapping‐induced wave amplitude modulation alone are shown by dashed‐dotted and dashed red vertical lines, respectively. The maximum length *β*_*ws*, max_ from Equation 5 of packets formed by wave superposition alone is indicated by a dashed blue vertical line. (d) Probability distributions of wave packets from Van Allen Probes statistical observations (black) and from the VHS code simulation during Single Wave intervals (red) and Two Waves intervals (blue), as a function of packet length *β*. (e) Probability distributions of wave packets from the VHS code simulation during Single Wave intervals (red) and Two Waves intervals (blue), as a function of packet peak amplitude *B*_*w*,*peak*_.

Figure 7 shows statistical results from this third VHS simulation, compared with statistical chorus wave packets observations from the Van Allen Probes. Here, there are only four long wave packets (with *β* ∼ 100–300, see Figure 7a) during Single Wave intervals of the simulation. PDFs of peak amplitudes *B*_*w*,*peak*_ of wave packets in the simulation during Single Wave periods (in red) and Two Waves intervals (in blue) are similar in Figure 7e, as in the previous simulations. In Figures 7c and 7d, the PDFs of wave packets obtained during Single Wave intervals (in red) of the simulation are totally different from the PDFs of chorus packets from statistical Van Allen Probes observations (in black): the latter peak at low *β* < 10 and $\beta \;{\left(B_{w,peak}/B_{0}\right)}^{1/2}<0.25$, whereas the former have a high maximum at *β* = 200–300 and $\beta \;{\left(B_{w,peak}/B_{0}\right)}^{1/2}=3-8$. In sharp contrast, PDFs of wave packets obtained during Two Waves intervals (in blue) of the simulation are nearly identical to PDFs of chorus packets from statistical Van Allen Probes observations, with peak occurrences at low *β* and $\beta \;{\left(B_{w,peak}/B_{0}\right)}^{1/2}$ values.

Figures 7a and 7d show that Single Wave intervals contain no short wave packet, whereas nearly 60% of the packets identified during Two Waves intervals are short (with *β* < 10). During Single Wave intervals of the simulation, 100% of the packets have lengths *β* > *β*_*tr*,*min*2_ > *β*_*tr*, min_ and very likely result from the nonlinear trapping‐induced amplitude modulation of a single wave. During Two Waves intervals, ∼90% of the packets have lengths *β* ≤ *β*_*ws*, max_ and can have been produced by wave superposition (see Figure 7c). Since ∼55% of the packets during Two Waves intervals have lengths *β* < *β*_*tr*, min_ and ∼87% have lengths *β* < *β*_*tr*,*min*2_, the majority of these packets cannot have been produced by trapping‐induced amplitude modulation alone. Normalized frequency sweep rates |*∂f*/*∂t*|/*f* ^2^ of wave packets in Figures 7a and 7b show a similar dependence |*∂f*/*∂t*|/*f* ^2^ ∼ 1/*β* ^2^ in observations and simulation, in agreement with dependencies due to nonlinear effects and wave superposition.

## 4 Selected Event of Conjugate Observations of Chorus Wave Packets and Electron Precipitation

In this section, we examine a selected event with THEMIS spacecraft observations of chorus wave packets and nearly conjugate ELFIN measurements of electron precipitation. The characteristics of chorus wave packets are first obtained from THEMIS data near the equator. Next, the observed characteristics of chorus wave packets are used, for the first time, to evaluate through test particle simulations the consequences of the fine structure of intense chorus waves on electron precipitation, allowing comparisons with electron precipitation measured by ELFIN at low altitude.

### 4.1 Chorus Wave Packets Observed in the Outer Belt

**Figure 8:**
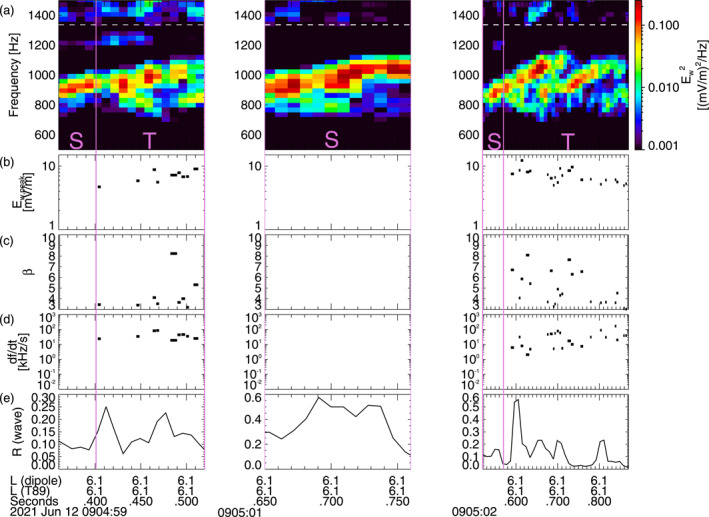
(a) Chorus wave Fast Fourier Transform (FFT) spectra from THEMIS E measurements on 12 June 2021 at *L* ≃ 6, approximately 1,000 km away from the magnetic equator. A white dashed line shows *f*_*ce*_/2. Intervals with only one single wave are marked by an S, intervals with two (or more) superposed waves are marked by a T, and these intervals are separated by vertical purple lines. (b) Peak amplitude of identified short packets, with a length *β* < 10. (c) Length *β* of short packets. (d) Frequency sweep rate *∂f*/*∂t* of short packets. (e) Average parallel to transverse wave electric field power ratio *R* of lower‐band chorus waves. The *L*‐shell values inferred from dipolar and T89 (Tsyganenko, 1989) magnetic field models are also provided.

Figure 8 shows three different intervals of lower‐band chorus wave measurements performed during a brief Burst mode period by the THEMIS E spacecraft (Angelopoulos, 2008) near 9:05 UT on 12 June 2021. THEMIS E was located at *L* ≃ 6 before dawn (5 MLT), near the equator (at magnetic latitudes |*λ*| < 3°). This event takes place during the recovery phase of a weak geomagnetic storm that reached a minimum *Dst* = −37 nT on the preceding day. Geomagnetic activity is moderate (*Kp* = 1.3–2) at 8–10 UT. Nevertheless, *Kp* earlier reached 4^−^ at 3–5 UT, suggesting the presence of substorm‐related injections of hot electrons from the plasma sheet generating the observed intense chorus waves (Li et al., 2010; Zhang et al., 2018). Survey mode data from THEMIS E, providing only four‐second averaged wave intensity, indicates similar chorus wave intensities between 7:00 UT and 9:10 UT as the spacecraft moved between *L* ∼ 5 and *L* ∼ 9, suggesting a similar chorus activity at *L* ∼ 6 during this whole period. But since Survey mode data cannot be used to analyze wave packets characteristics, we have to rely on the waveform data analyzed in Figure 8 to estimate chorus packets characteristics near *L* = 6 during this event. Fortunately, such characteristics will appear (see below) as typical of lower‐band chorus packets in Van Allen Probes statistics, lending confidence that the measured values are representative of chorus packets present during this whole period.

We use chorus wave measurements from THEMIS three‐axis antennas of the electric field instrument (EFI; Bonnell et al., 2008), and background magnetic field measurements by the fluxgate magnetometer (Auster et al., 2008). The plasma density is inferred from the spacecraft potential and electron thermal speed, respectively provided by EFI and the electro‐static analyzer (Bonnell et al., 2008; McFadden et al., 2008). The observed lower‐band chorus waves are mostly quasi‐parallel waves, with a measured parallel to transverse electric field power ratio *R* < 0.2–0.3 most of the time in Figure 8 (e.g., see Artemyev et al., 2016). We use the same threshold *B*_*threshold*_ = 50 pT as in Section 3 to determine wave packets limits, but converted to an electric field threshold *E*_threshold_ = *B*_threshold_ × 0.3/*N* ≃ 1.8 mV/m, with *N* the wave refractive index calculated based on wave frequency, plasma density and gyrofrequency provided by THEMIS instruments.

During this event, THEMIS E observed similar chorus wave elements as in VHS simulations presented in Section 3, with an average frequency *ω*/Ω_*ce*_ ∼ 0.35. The first time period in Figure 8 contains a long rising tone together with a short falling tone. The second time period contains only a rising tone. The third time period contains several long rising tone elements occurring in close succession, which favors superposition of successive elements. During this whole event, most chorus waves had much the same characteristics as waves in the third time period. These different types of chorus elements are similar to chorus elements obtained in VHS simulations in Section 3. In THEMIS observations, Single Wave intervals are found near the start or middle/end of long rising tone chorus elements, while Two Waves intervals occur at the end or beginning of long chorus elements, corresponding to the formation of short packets with *β* = 3–8 and large frequency sweep rates |*∂f*/*∂t*| = 20–200 kHz/s, as in simulations. Peak amplitudes *E*_*w*,*peak*_ of wave packets are comprised between 5 and 15 mV/m, corresponding to ∼100–300 pT. Such wave packet characteristics are similar to typical characteristics of chorus wave packets in Van Allen Probes statistics at *L* ≃ 4–6 (Zhang, Mourenas, et al., 2020; Zhang et al., 2018).

Although the measured frequency separation Δ*f* between simultaneous chorus waves is smaller (Δ*f* ∼ 100–200 Hz) during these THEMIS observations than in VHS simulations in Section 3, Δ*f*/*f* remains similar, because the electron gyrofrequency *f*_*ce*_ and lower‐band chorus frequencies are both roughly twice smaller during this event at *L* = 6 than in the simulations performed for *L* = 5. Very weak upper‐band chorus waves are sometimes present near 1.5 kHz in Figure 8, but with much smaller amplitudes than lower‐band chorus waves.

During this event of weak geomagnetic activity (with −10 nT ≤ *Dst* ≤ −5 nT and 1.3 ≤ *Kp* ≤ 2) at 8:00–9:10 UT, the geomagnetic field configuration remained dipolar at THEMIS's location near 5 MLT and *L* ≃ 6, with identical *L* values inferred from dipolar and disturbed T89 magnetic field models in Figure 8. However, it is worth noting that during more active periods the magnetic field can become strongly stretched near midnight, which can modify chorus wave generation, frequency sweep rate, and chorus element duration (Katoh & Omura, 2013; Tao et al., 2014; Teng et al., 2017).

**Figure 9:**
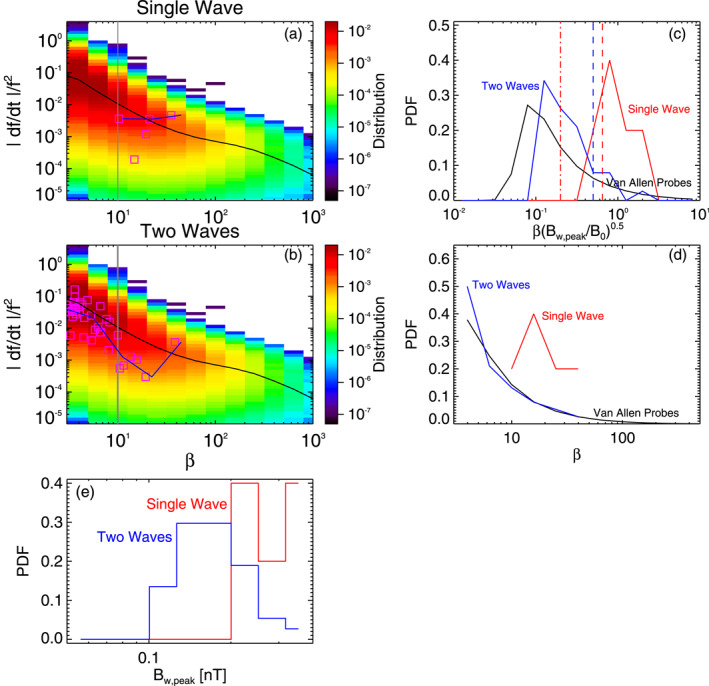
(a) THEMIS E observations of lower‐band chorus wave packets at *L* = 6 on 12 June 2021, during Single Wave intervals in Figure 8. Normalized frequency sweep rate |*∂f*/*∂t*|/*f* ^2^ of wave packets from THEMIS measurements in the generation region near the equator (magenta squares) as a function of packet length *β* (the median is shown by a blue curve). Corresponding statistical results from Van Allen Probes 2012–2018 observations are displayed in colors, with their median |*∂f*/*∂t*|/*f* ^2^ shown by a black curve. A gray line shows *β* = 10. (b) Same as (a) during Two Waves intervals. (c) Probability Distribution Function of wave packets as a function of $\beta \;{\left(B_{w,peak}/B_{0}\right)}^{1/2}$ obtained from Van Allen Probes statistical observations (black curve) and from THEMIS E measurements during Single Wave intervals (red) and Two Waves intervals (blue). The minimum lengths *β*_*tr*, min_ and *β*_*tr*,min2_ from Equations 2 and 3 of wave packets formed by nonlinear trapping‐induced wave amplitude modulation alone are shown by dashed‐dotted and dashed red vertical lines, respectively. The maximum length *β*_*ws*, max_ from Equation 5 of packets formed by wave superposition alone is indicated by a dashed blue vertical line. (d) Probability distributions of wave packets from Van Allen Probes statistical observations (black) and from THEMIS observations during Single Wave intervals (red) and Two Waves intervals (blue), as a function of packet length *β*. (e) Probability distributions of wave packets from THEMIS measurements during Single Wave intervals (red) and Two Waves intervals (blue), as a function of packet peak amplitude *B*_*w*,*peak*_.

Figure 9 shows statistical results from this event, compared with statistical chorus packet observations from the Van Allen Probes. The PDFs of the peak amplitudes *B*_*w*,*peak*_ of wave packets measured by THEMIS during Single Wave intervals (in red) and Two Waves intervals (in blue) are relatively similar in Figure 9e, except for ∼50% higher peak amplitudes during Single Wave intervals. In Figures 9c and 9d, the PDFs of wave packets measured by THEMIS during Single Wave intervals (in red) are very different from the PDFs of chorus packets from statistical Van Allen Probes observations (in black). The PDFs of chorus packets from statistical observations peak at low *β* < 10 and $\beta \;{\left(B_{w,peak}/B_{0}\right)}^{1/2}<0.25$, whereas the PDFs of chorus packets measured by THEMIS during Single Wave intervals peak at *β* = 15 and $\beta \;{\left(B_{w,peak}/B_{0}\right)}^{1/2}=0.5-2.0$. Conversely, the PDFs of wave packets measured by THEMIS during Two Waves intervals (in blue) are very similar to the PDFs of chorus packets from statistical Van Allen Probes observations, with peak occurrences at low *β* and $\beta \;{\left(B_{w,peak}/B_{0}\right)}^{1/2}$. Figures 9a and 9d show that Single Wave intervals contain no short wave packet (with *β* < 10), whereas 70% of the packets identified during Two Waves intervals are short. During Single Wave intervals, 80% of the packets are such that *β* > *β*_*tr*,min2_ and 100% have *β* > *β*_*tr*, min_, indicating that they likely result from a nonlinear trapping‐induced wave amplitude modulation. Conversely, during Two Waves intervals, 90% of the packets have lengths *β* ≤ *β*_*ws*, max_ and can have been produced by wave superposition (see Figure 9c). It is worth emphasizing that during Two Waves intervals, ∼50% of the packets have lengths *β* < *β*_*tr*, min_ and ∼90% have lengths *β* < *β*_*tr*,*min*2_, showing that at least half of these packets cannot have been produced by trapping‐induced amplitude modulation alone.

Normalized frequency sweep rates |*∂f*/*∂t*|/*f* ^2^ of short chorus wave packets (with *β* < 10) in Figure 9b show a roughly similar dependence |*∂f*/*∂t*|/*f* ^2^ ∼ 1/*β* ^2^ in THEMIS observations on 12 June 2021 and in statistical observations from the Van Allen Probes in 2012–2018, in agreement with the expected dependence for a prevalent wave superposition mechanism. Long wave packets are too rare during this event to get a precise estimate of the |*∂f*/*∂t*|/*f* ^2^ dependence on *β*, but they do remain within the range of highest occurrences of Van Allen Probe statistics, shown in red‐orange in Figure 9b. Therefore, Figure 9 shows that chorus wave packets observed by THEMIS during this selected event are representative of typical chorus packets, and are also similar to packets obtained in VHS simulations in Section 3. Finally, it is worth emphasizing that a realistic simulation of nonlinear chorus wave generation near the equator, performed with the Electron Hybrid code (which treats cold electrons as a fluid and energetic electrons as particles via the particle‐in‐cell method) has produced two long chorus rising tones occurring in very close succession (Katoh & Omura, 2016), as in the present THEMIS observations, forming a lot of short wave packets with similar statistical characteristics as in THEMIS observations in Figure 9 (Zhang et al., 2021).

### 4.2 Impact of Short Chorus Wave Packets on Electron Precipitation

During the 12 June 2021 event discussed in Section 4.1, chorus wave observations near the geomagnetic equator by THEMIS E in Burst mode near 9:04 UT at *L* ≃ 6 (and in Survey mode at *L* ∼ 5–6 and 4–5 MLT between 7:00 UT and 9:10 UT) can be supplemented by nearly conjugate observations of electron precipitation, provided by the two low‐altitude ELFIN A & B CubeSats on polar orbits (Angelopoulos et al., 2020). At 8:20 UT and 8:58 UT, ELFIN A or B crossed *L* = 6 at 2.0–2.5 MLT, less than 2–3 hr earlier in MLT than THEMIS E, and in the latest case within 6 min (UT) of THEMIS E measurements displayed in Figure 8. Note that similar chorus waves are usually generated over the 2–5 MLT sector by injected anisotropic electron populations during periods with *Kp* = 1.3–2 (Agapitov et al., 2018; Li et al., 2010; Tao et al., 2011), in agreement with the typical correlation scale ΔMLT ≈ 2 hr over 2–5 MLT of the chorus source region at *L* ≃ 6 (Agapitov et al., 2021). This is confirmed during this event by THEMIS E Survey mode data showing similar chorus waves at *L* = 5–6.3 and 4–5 MLT during the whole 8:00–9:10 UT time interval. Therefore, it is reasonable to assume that lower‐band chorus waves similar to the waves measured in Burst mode by THEMIS E at *L* ≃ 6 and 9:04 UT near 5 MLT were also present at 8:19 UT and 8:58 UT (i.e., less than 45 min and less than 6 min earlier) near 2–2.5 MLT, when ELFIN CubeSats recorded the corresponding electron precipitation.

ELFIN A & B CubeSats, on low‐Earth orbit at ∼450 km, measure electron fluxes between 50 keV and 5 MeV, with a full resolution of electron pitch angles over each spin period (∼3 s) of the spacecraft transverse to the orbit plane, enabling measurements of the flux *J*_*trapped*_ of trapped/quasi‐trapped electrons, the flux *J*_*precip*_ of precipitating electrons averaged within the loss cone, and the upward flux *J*_*up*_ of electrons backscattered by the atmosphere averaged within the loss cone, with a high signal‐to‐noise ratio of about 50:1 in a typical electron and ion environment at *L* ∼ 5–6 (Angelopoulos et al., 2020; Mourenas et al., 2021).

**Figure 10:**
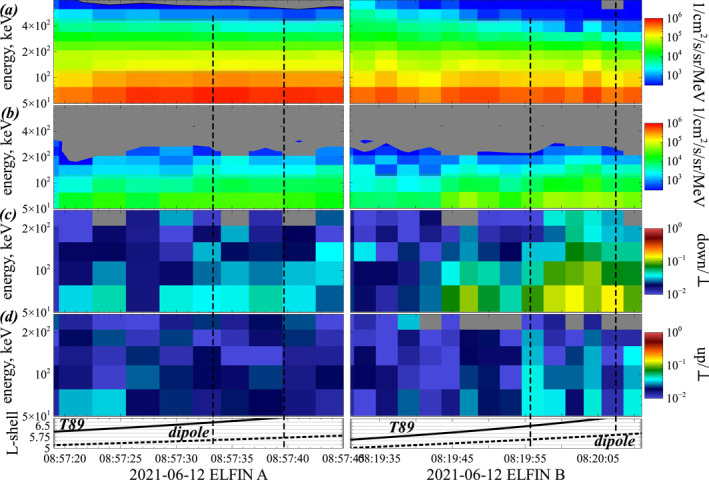
ELFIN A & B low‐altitude measurements of electron fluxes at *L* ∼ 5.5–6.5 and 2 MLT, on 12 June 2021. (a) Trapped electron flux *J*_*trapped*_ at *α* ≃ 1.05*α*_*LC*_ with *α*_*LC*_ the loss cone angle, (b) precipitating electron flux *J*_*precip*_ (averaged over the loss cone), (c) *J*_*precip*_/*J*_*trapped*_, (d) ratio *J*_*up*_/*J*_*trapped*_ of upward flux backscattered within the loss cone to trapped flux. Gray regions in panels (a, b) correspond to instrument noise level (∼3 counts/s). Vertical dashed lines mark the start and end of two intervals of intense and modulated (likely wave‐induced) precipitation. During this weakly disturbed period with *Kp* = 1.3, the actual *L*‐shell was likely between values derived from dipolar and T89 (Tsyganenko, 1989) geomagnetic fields.

Figure 10 displays trapped electron fluxes *J*_*precip*_, and ratios *J*_*precip*_/*J*_*trapped*_ and *J*_*up*_/*J*_*trapped*_ measured by ELFIN A & B in the outer radiation belt at *L* = 5.5–6.3 and 2 MLT near 8:20 UT and 8:58 UT during this event, when both CubeSats were on geomagnetic field lines west from the South Atlantic Anomaly region. Precipitation of 50–200 keV electrons is continuously observed at *L* ≃ 6, with modulations of precipitating flux over timescales of 5–15 s. The precipitating to trapped electron flux ratio reaches significant levels *J*_*precip*_/*J*_*trapped*_ ∼ 0.05–0.1 up to 100–200 keV, indicating that such electrons are efficiently scattered in pitch angle toward the loss cone through resonant interactions with the intense lower‐band chorus waves observed by THEMIS E (Kasahara et al., 2018; Kubota & Omura, 2018; Mourenas et al., 2021). However, the observed flux ratio *J*_*precip*_/*J*_*trapped*_ ∼ 0.05–0.1 remains moderate, suggesting that it may still correspond to a nearly diffusive (quasi‐linear‐like) transport of electrons toward the loss cone (Kennel & Petschek, 1966; Li et al., 2013; Mourenas et al., 2021). The upward electron flux *J*_*up*_ backscattered by the atmosphere inside the loss cone is significant, with *J*_*up*_/*J*_*precip*_ ∼ 1/3. As in previous work (Mourenas et al., 2021), we assume a quasi‐steady‐state system nearly symmetric about the equator over time intervals much longer than the electron bounce period *τ*_*B*_ ∼ 1 s, with similar average fluxes of 50–200 keV electrons backscattered inside the loss cone by the atmosphere in both hemispheres (see also Selesnick et al., 2004). Accordingly, the net time‐averaged electron flux $J_{\mathrm{precip}}^{\mathrm{net}}$ directly precipitated by chorus waves is equal to the measured average precipitating flux within the loss cone *J*_*precip*_, minus the average backscattered flux within the loss cone coming from the opposite hemisphere *J*_*back,opp*_ ∼ *J*_*up*_, finally giving $J_{\mathrm{precip}}^{\mathrm{net}}≃J_{\mathrm{precip}}-J_{up}$ (Mourenas et al., 2021).

Similar electron precipitation patterns were observed by ELFIN from 6:47 UT to 8:58 UT around *L* = 6, attesting the presence of lower‐band chorus waves with similar properties during this whole period. Two typical intervals of 6–12 s of precipitation measured by ELFIN at *L* ∼ 6, delimited by vertical dashed lines in Figure 10, have been selected during this event, to provide time‐averaged precipitated and trapped electron fluxes representative of the whole event. Hereafter, we use test particle simulations to reproduce such ELFIN observations of precipitating electron fluxes driven by resonant interactions with chorus waves measured by THEMIS E. The test particle simulation code used has already been described in a previous article (Zhang, Agapitov, et al., 2020).

THEMIS E measurements (Angelopoulos et al., 2008; Auster et al., 2008; Bonnell et al., 2008; McFadden et al., 2008) near the equator provide the trapped flux *J*_*THE*_(*E*, *α*), the plasma frequency to gyrofrequency ratio Ω_*pe*_/Ω_*ce*_ ∼ 5 at *L* ∼ 6 near 8:58 UT, the average peak wave amplitude *B*_*w*,*peak*_ ∼ 160 pT, the typical wave packet length *β* ∼ 5 (corresponding to ∼5 ms), and the average quasi‐parallel wave frequency *ω*/Ω_*ce*_ = 0.35 (see Figures 8 and 9), used as initial conditions in simulations. Some wave packets are longer or more intense, with *β* ∼ 20 (lasting ∼20 ms) and *B*_*w*,*peak*_ ∼ 250 pT, but they are rare. In test particle simulations, the distance between wave packets is taken equal to packet length. In this case, particles near the loss cone that escape from resonant trapping (Artemyev, Neishtadt, Albert, et al., 2021; Kitahara & Katoh, 2019) usually cannot be trapped by the next packet, which is roughly equivalent to having large random phase jumps between packets as in chorus wave statistics (Zhang, Agapitov, et al., 2020)—a situation roughly equivalent to considering independent wave packets. All the above parameters are consistent with statistical observations in the dawn sector at *L* ∼ 6 during similar moderately disturbed periods (Agapitov et al., 2018; Mourenas et al., 2021; Sheeley et al., 2001; Zhang, Mourenas, et al., 2020; Zhang et al., 2018).

In a first series of simulations, lower‐band chorus wave amplitudes are assumed to remain constant from the equator up to a latitude of *λ* ∼ 40°, as in statistical observations at *L* = 6 and 0–3 MLT during quiet periods with *Kp* < 1 (Agapitov et al., 2018). Such an unattenuated propagation of intense lower‐band chorus waves up to middle‐to‐high latitudes can be allowed by a weak Landau damping during quiet periods, when waves propagate guided inside high‐density ducts (Artemyev, Demekhov, et al., 2021; Chen et al., 2013; Hosseini et al., 2021; Ke et al., 2021; Streltsov et al., 2012). Simulations are also performed with a more realistic latitudinal distribution of the wave amplitude, decreasing at *λ* > 5° like $g\left(\lambda \right)=\tanh\left({\left(\lambda /2{}^{\circ}\right)}^{2}\right)\cdot \exp\left(-{\left(\lambda /20{}^{\circ}\right)}^{2}\right)$, as in statistical observations at *L* = 6 and 0–3 MLT during moderately disturbed periods with *Kp* ≃ 1.3–2 as here (Agapitov et al., 2018). This decrease of the wave amplitude along its propagation to higher latitudes is due to Landau damping (Chen et al., 2013). Additional simulations are performed with very long wave packets (*β* → ∞).

Between 8:00 UT and 9:10 UT on 12 June 2021, the geomagnetic activity remained weak, with −10 nT ≤ *Dst* ≤ −5 nT and 1.3 ≤ *Kp* ≤ 2. This should correspond to only weak variations in the geomagnetic field configuration at *L* ≃ 6 and 2–5 MLT as compared with a dipolar field, as confirmed by the roughly similar *L*‐shell positions of ELFIN inferred from undisturbed dipolar and disturbed T89 (Tsyganenko, 1989) geomagnetic field models in Figures 8 and 10 (taking into account that deviations from a dipolar field are often overestimated by the T89 model near midnight, see McCollough et al., 2008). This justifies approximating the real geomagnetic field by a dipolar field to first order in test‐particle simulations.

**Figure 11:**
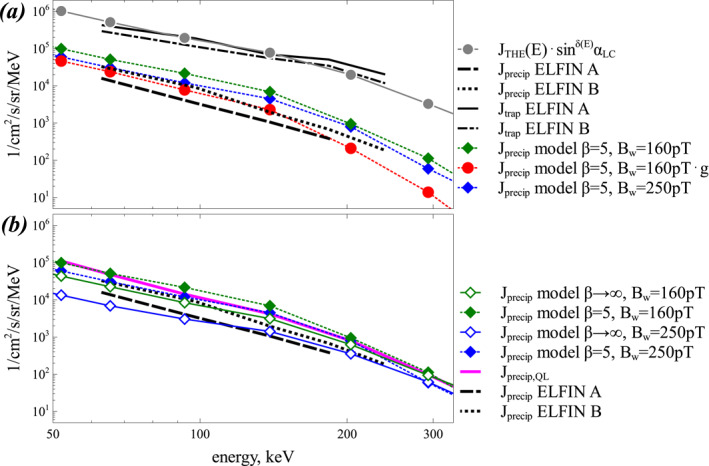
Results from test particle simulations based on plasma and chorus wave packet properties measured by THEMIS E at *L* ≃ 6 and 5 MLT near 9 UT, compared with contemporaneous low‐altitude ELFIN observations at *L* ≃ 6 and 2 MLT on 12 June 2021. (a) Trapped electron flux *J*_*trapped*_ at *α* ≃ 1.05*α*_*LC*_ measured by ELFIN A & B (solid and dashed‐dotted black lines), and used in simulations (gray circles). Net precipitating electron flux $J_{\mathrm{precip}}^{\mathrm{net}}$ (averaged within the loss cone) measured by ELFIN A & B (dashed and dotted black curves) and obtained from simulations (symbols). Simulations are performed for *β* = 5, with a constant *B*_*w*,*peak*_ = 160 pT (green diamonds), a constant *B*_*w*,*peak*_ = 250 pT (blue diamonds), or with *B*_*w*,*peak*_ = 160 ⋅ *g*(*λ*) pT (red circles). A constant *B*_*w*,*peak*_(*λ*) corresponds to statistical observations when *Kp* < 1, while function $g\left(\lambda \right)=\tanh\left({\left(\lambda /2{}^{\circ}\right)}^{2}\right)\cdot \exp\left(-{\left(\lambda /20{}^{\circ}\right)}^{2}\right)$ fits the statistical variation of chorus wave amplitude away from the equator when *Kp* ≃ 1.3–2, notably its significant decrease at latitudes *λ* > 5° (Agapitov et al., 2018). To estimate the occurrence rate of intense wave packets in simulations, we impose the same time‐averaged wave intensity $\langle B_{w}^{2}\left(t\right)\rangle =70^{2}$ pT^2^ at *λ* ∼ 2°–5° as in THEMIS observations. (b) Same as (a) but showing only *J*_*precip*_ measured by ELFIN, and *J*_*precip*_ from simulations with a constant *B*_*w*,*peak*_ = 160 pT (green) or 250 pT (blue), for *β* = 5 (filled diamonds) and *β* → *∞* (empty diamonds), and the quasi‐linear estimate *J*_*precip,QL*_ for a constant *B*_*w*_ (solid magenta line).

During this event, the short intense chorus wave packets measured by THEMIS E (see Figure 8) belong to long rising tones lasting ∼0.2 s, separated by intervals of weaker (or null) wave power. Therefore, the propagating intense wave packets are not continuously present at a given latitude of cyclotron resonance with electrons during a bounce period *τ*_*B*_ ∼ 1 s of 50–200 keV electrons along a geomagnetic field line. Accordingly, the occurrence rate of intense wave packets is adjusted in test particle simulations, so that the time‐averaged wave intensity near the equator be equal to the nearly constant time‐averaged intensity $E_{w}^{2}∼2.5^{2}$ mV^2^/m^2^ (i.e., $B_{w}^{2}∼70^{2}$ pT^2^) of lower‐band chorus waves measured by THEMIS E at *L* ≃ 6 during the 6 s burst mode period near 9:00 UT on 12 June 2021 investigated in Figures 8 and 9. For consistency with both ELFIN and THEMIS E measurements, the trapped electron flux *J*_*THE*_(*E*) measured near the equator by THEMIS E at high equatorial pitch angles *α* ≈ 90° is multiplied by a function sin^*d*(*E*)^ *α* (where *d*(*E*) is twice the pitch angle anisotropy index *s* of the electron distribution, see Summers et al., 2009) to approximately recover the trapped flux *J*_*trapped*_(*E*) measured by ELFIN immediately above the bounce loss cone angle *α*_*LC*_ (see Figure 11a) during the two typical precipitation intervals shown in Figure 10. At the start of the simulations, *J*(*E*, *α*) = 0 at *α* < *α*_*LC*_.

Results from our test particle simulations are displayed in Figure 11 (see numerical scheme details in Zhang, Agapitov, et al., 2020). 10^5^ electron trajectories are calculated for each set of parameters. First, trajectories of initially trapped electrons (initially outside the loss cone) with a distribution *J*_*trapped*_(*E*, *α*) = *J*_*THE*_(*E*) sin^*d*(*E*)^ *α* are calculated along the *L* = 6 magnetic field line. Next, the phase space density inside the loss cone is calculated after one bounce period $\tau_{B}∼0.66\gamma /{\left(\gamma^{2}-1\right)}^{1/2}$ s (with γ the Lorentz factor) of particles along the geomagnetic field line *L* = 6 (Schulz & Lanzerotti, 1974), providing the precipitating electron flux *J*_*precip*_ averaged within the loss cone.

In Figure 11a, the energy spectrum of precipitating electron fluxes *J*_*precip*_(*E*) measured by ELFIN A & B (dashed and dotted black curves) is well reproduced by simulations with realistic chorus wave packet parameters, that is, with *β* = 5 and a wave amplitude decreasing as *B*_*w*,*peak*_ = 160 ⋅ *g*(*λ*) pT (red circles) at latitudes *λ* > 5° due to Landau damping (red circles). The precipitating fluxes measured by ELFIN B at 8:19 UT are slightly higher than precipitating fluxes measured by ELFIN A at 8:57 UT, likely due to spatio‐temporal fluctuations of chorus wave power. In contrast, simulations performed with *β* = 5 and a constant wave amplitude *B*_*w*,*peak*_ = 160 pT (green filled diamonds) give significantly higher precipitating fluxes than in ELFIN observations, due to an overestimation of the wave amplitude at middle latitudes *λ* ∼ 14°–25° where cyclotron resonance with 60–250 keV electrons occurs (Agapitov et al., 2018; Artemyev et al., 2016). This confirms the likely presence of a significant Landau damping of lower‐band chorus waves by keV electrons (Chen et al., 2013), leading to a reduction of the wave amplitude by a factor *g* ∼ 0.6 to *g* ∼ 0.2 from *λ* ∼ 14° to *λ* ∼ 25° during this event.

Interestingly, simulations with *β* = 5 give a slightly smaller *J*_*precip*_ at all energies for *B*_*w*,*peak*_ = 250 pT (blue diamonds) than for *B*_*w*,*peak*_ = 160 pT (green diamonds), although both simulations have the same time‐averaged wave intensity $\langle B_{w}^{2}\rangle$. This is due to the slower increase of *D*_*αα*_ with *B*_*w*_ in test particle simulations compared to quasi‐linear diffusion (which scales as $D_{\alpha \alpha }∼\langle B_{w}^{2}\rangle$) above a threshold *B*_*w*_ ∼ 10^−3^ *B*_0_ ∼ 150 pT corresponding to the transition between quasi‐linear diffusion and nonlinear resonant scattering (Tao et al., 2012). Indeed, this leads to a ratio *D*_*αα*_ (*B*_*w*,*peak*_ = 250pT)/*D*_*αα*_ (*B*_*w*,*peak*_ = 160pT) < (250/160)^2^ in test particle simulations when such wave packets are present. But 250 pT wave packets are present a fraction of time Δ*t*_250_/Δ*t*_160_ = (160/250)^2^ less than 160 pT waves to keep the same time‐averaged wave intensity $\langle B_{w}^{2}\rangle$ in both simulations. This finally gives a net ratio of time‐averaged scattering rates during these two simulations of 〈*D*_*αα*_ (*B*_*w*,*peak*_ = 250 pT)〉/〈*D*_*αα*_ (*B*_*w*,*peak*_ = 160 pT)〉 = [*D*_*αα*_ (*B*_*w*,*peak*_ = 250 pT)/*D*_*αα*_ (*B*_*w*,*peak*_ = 160 pT)] × Δ*t*_250_/Δ*t*_160_ < 1, leading to a smaller *J*_*precip*_ for simulations with *B*_*w*,*peak*_ = 250 pT than for *B*_*w*,*peak*_ = 160 pT.

Finally, results from test particle simulations performed with a constant *B*_*w*,*peak*_ and short wave packets are compared in Figure 11b with results from quasi‐linear diffusion theory, and with results from simulations performed with very long packets. Albert (2010) has shown that electron interactions with quasi‐parallel monochromatic waves in an inhomogeneous magnetic field cause particle diffusion with a corresponding narrowband spectrum quasi‐linear diffusion coefficient identical to the diffusion coefficient obtained in the usual limit of a broadband spectrum (see also Mourenas et al., 2012), allowing in principle to use the quasi‐linear diffusion theory to model chorus wave‐particle interactions in the limit of not too high wave amplitudes (as verified in simulations by Tao et al., 2012). This justifies comparing the results of the present test particle simulations with results from quasi‐linear diffusion theory.

During this event at *L* = 6 (where Ω_*pe*_/Ω_*ce*_ = 5), the observed quasi‐parallel chorus waves of average intensity $B_{w}^{2}∼70^{2}$ pT^2^ and frequency *ω*/Ω_*ce*_ = 0.35 correspond to a quasi‐linear pitch angle diffusion rate *D*_*αα*,*LC*_ ∼ 2.3 × 10^−4^ s^−1^ at *α*_*LC*_ for 100 keV electrons (Artemyev et al., 2016; Mourenas et al., 2014). For strictly quasi‐linear diffusion, the precipitating (at *α* < *α*_*LC*_) to trapped (at *α* ≃ 1.05*α*_*LC*_) electron flux ratio (Kennel & Petschek, 1966) can be formulated as *J*_*precip*_(*α*)/*J*_*trapped*_ ∼ exp(−*z*_0_ *δα*)/(1 + *z*_0_/20) for sufficiently large $z_{0}=2\;\alpha_{LC}/\sqrt{D_{\alpha \alpha ,LC}\tau_{B}}>4$ (Mourenas et al., 2021), with *δα* = (1 − *α*/*α*_*LC*_) ∈ [0, 1] and *α* the equatorial pitch angle in radians. The corresponding average precipitating flux within the loss cone is $J_{\mathrm{precip,QL}}∼J_{\mathrm{trapped}}/\left(z_{0}+z_{0}^{2}/20\right)$. Using the analytical scaling of *D*_*αα*,*LC*_ with electron energy (Mourenas et al., 2014), we get $z_{0}≃23\;{\left(E+E^{2}\right)}^{5/8}$, with *E* in MeV, for a constant wave amplitude *B*_*w*_ = 70 pT.

Figure 11b shows that *J*_*precip*_ obtained from the simulation with *β* = 5 and a constant *B*_*w*,*peak*_ = 160 pT (with an occurrence rate of wave packets adjusted to have a time‐averaged wave intensity $\langle B_{w}^{2}\left(t\right)\rangle =70^{2}$ pT^2^) remains in good agreement with *J*_*precip,QL*_ over more than three decades of precipitating flux between 50 and 330 keV (compare green filled diamonds and solid magenta curve). This confirms that resonant interactions with independent, short, and moderately intense chorus wave packets essentially correspond to a regime of quasi‐linear electron diffusion (Artemyev, Neishtadt, Vasiliev, et al., 2021; Mourenas et al., 2018; Zhang, Agapitov, et al., 2020).

Simulations with very long packets (*β* → ∞) lead to a much smaller precipitating flux *J*_*precip*_ below 150 keV than simulations with short packets (*β* = 5) in Figure 11b, for both *B*_*w*,*peak*_ = 160 pT and *B*_*w*,*peak*_ = 250 pT (compare empty and filled diamonds). This is due to an important nonlinear process, called anomalous trapping. Anomalous trapping can strongly increase the pitch angle of low energy electrons with initially small pitch angles (Artemyev, Neishtadt, Albert, et al., 2021; Gan et al., 2020; Kitahara & Katoh, 2019), preventing their final precipitation and consequently decreasing *J*_*precip*_. But the efficiency of anomalous trapping is strongly reduced in the case of short packets, because electrons escape much faster from trapping in short packets (see Appendix in Mourenas et al., 2021). Below 150 keV, the stronger efficiency of anomalous trapping for *β* → ∞ than for *β* = 5 explains the smaller *J*_*precip*_ obtained in simulations with *β* → ∞ than in simulations with *β* = 5.

For *β* → ∞, anomalous trapping is also more effective for waves of higher amplitude (Artemyev, Neishtadt, Albert, et al., 2021; Kitahara & Katoh, 2019). Below 150 keV, this leads to a ∼3 times smaller *J*_*precip*_ for *B*_*w*,*peak*_ = 250 pT than for *B*_*w*,*peak*_ = 160 pT (compare blue and green empty diamonds). In contrast, simulations performed with *β* = 5 and *B*_*w*,*peak*_ = 250 pT (blue filled diamonds) yield a similar *J*_*precip*_ as for a lower peak amplitude *B*_*w*,*peak*_ = 160 pT (green filled diamonds), close to *J*_*precip,QL*_. This demonstrates that the actual fine structure of intense chorus rising tones, consisting of many short packets/subpackets, significantly reduces nonlinear effects, leading to a nearly quasi‐linear diffusive evolution where the *J*_*precip*_/*J*_*trapped*_ ratio is mainly controlled by the time‐averaged wave intensity, fixed here at the same level in all simulations (Artemyev, Neishtadt, Vasiliev, et al., 2021; Mourenas et al., 2021, 2018; Zhang, Agapitov, et al., 2020).

Compared with simulations performed with *β* = 5 and *B*_*w*,*peak*_ = 160 ⋅ *g*(*λ*) pT, simulations performed with *β* → *∞* and a constant *B*_*w*,*peak*_ = 160 pT (assuming ducted wave propagation) give *J*_*precip*_ values as close to ELFIN observations at 65–90 keV, but farther from ELFIN values at 130–230 keV (see Figures 11a and 11b). Including a realistic decrease *g*(*λ*) of the wave amplitude toward middle latitudes (Agapitov et al., 2018; Ke et al., 2021) in simulations with *β* → ∞ would bring their *J*_*precip*_ values farther from ELFIN values. Therefore, simulations performed with realistic chorus wave packet parameters, *β* = 5 and *B*_*w*,*peak*_ = 160 ⋅ *g*(*λ*) pT, provide the most consistent explanation for the precipitating fluxes measured by ELFIN.

## 5 Conclusions

Short wave packets are ubiquitous in Van Allen Probes statistics of intense lower‐band chorus waves in the outer radiation belt. In this article, we first checked with new VHS code simulations, performed with one or two triggering waves, that the length of such short wave packets is consistent with a criterion of resonance non‐overlap for two independent superposed waves. Separating simulation results in Single Wave and Two Waves intervals based on spectral characteristics, we found that Two Waves intervals mainly contain short chorus wave packets (*β* < 10) with large frequency sweep rates *∂f*/*∂t* ∼ 40–400 kHz/s, likely produced by wave superposition and with similar statistical characteristics as in 2012–2018 Van Allen Probes observations. In contrast, Single Wave intervals mostly contain long chorus wave packets with moderate sweep rates, likely produced by nonlinear trapping‐induced wave amplitude modulation. The dependence of the sweep rate *∂f*/*∂t* on packet length *β* is similar in simulations and Van Allen Probes statistics. The results from these new VHS simulations therefore strengthen the conclusions previously drawn by Nunn et al. (2021) from two VHS simulations performed with different initial parameters. In addition, we found that short chorus wave packets are mainly formed near the middle/end of long rising tones for moderate linear growth rates, and everywhere for strong linear growth rates.

Next, we similarly analyzed an event of long chorus rising tone elements observed by the THEMIS E spacecraft at *L* = 6 near the magnetic equator on 12 June 2021, finding similar characteristics of Single Wave and Two Waves intervals as in VHS simulations and 2012–2018 Van Allen Probes statistics. During this event, simultaneous observations by ELFIN CubeSats of precipitating electron fluxes at low altitude in the same (*L*, MLT) sector allowed us to investigate the role played by short chorus wave packets in energetic electron precipitation.

The precipitating electron fluxes measured by ELFIN at low altitude have been well recovered by test particle simulations performed using the characteristics of plasma and lower‐band chorus wave packets measured by THEMIS near the equator, namely mostly short (*β* ∼ 5) wave packets of moderate peak amplitudes *B*_*w*,*peak*_ ∼ 160 pT, combined with a reduction of wave amplitude by Landau damping during wave propagation to middle latitudes, taken from statistical observations at *L* ∼ 6 and 0–3 MLT during moderately disturbed periods (Agapitov et al., 2018).

We also found that chorus wave‐driven electron precipitation is significantly different for short and long wave packets. Short wave packets essentially lead to a more diffusive‐like transport of 50–200 keV electrons toward the loss cone than very long packets, as previously noted in the case of electron energization at higher pitch angles (Artemyev, Neishtadt, Vasiliev, et al., 2021; Zhang, Agapitov, et al., 2020). In such a case, the main parameter determining the precipitating to trapped electron flux ratio *J*_*precip*_/*J*_*trapped*_ is the time‐averaged wave intensity, as in the quasi‐linear diffusion paradigm (Kennel & Petschek, 1966). In contrast, very long wave packets lead to a more nonlinear electron transport, where anomalous trapping (Albert et al., 2021; Artemyev, Neishtadt, Albert, et al., 2021; Gan et al., 2020; Kitahara & Katoh, 2019) significantly reduces electron precipitation below 150 keV, an effect which becomes more marked for wave packets of higher peak amplitudes *B*_*w*,*peak*_ > 200 pT.

## Data Availability

Open ResearchVan Allen Probes EMFISIS data is available at https://emfisis.physics.uiowa.edu/data/, THEMIS data is available at http://themis.ssl.berkeley.edu, and ELFIN data is available at http://themis-data.igpp.ucla.edu/ela/. Data access and processing was done using the SPEDAS V4.1 software (Angelopoulos et al., 2019) available at https://spedas.org. The test particle simulation code used here has been described in a previous article (Zhang, Agapitov, et al., 2020).
